# Identification of novel collagen breakdown products by human osteoclasts in vitro and in vivo

**DOI:** 10.1093/jbmrpl/ziaf160

**Published:** 2025-10-11

**Authors:** Brendan P Norman, Jane P Dillon, Sahem M Alkharabsheh, Francoise Congues, Peter J M Wilson, Andrew S Davison, Elinor Chapman, James Baker, Séamus Coyle, Chris Probert, Paul E Beaulé, Lakshminarayan R Ranganath, Jeremy M Wilkinson, James A Gallagher

**Affiliations:** Department of Musculoskeletal and Ageing Science, Institute of Life Course and Medical Sciences, University of Liverpool, West Derby Street, Liverpool, L7 8TX, United Kingdom; Department of Musculoskeletal and Ageing Science, Institute of Life Course and Medical Sciences, University of Liverpool, West Derby Street, Liverpool, L7 8TX, United Kingdom; Department of Medical Laboratory Sciences, Faculty of Allied Medical Sciences, Mutah University, Alkarak 61710, Jordan; Department of Musculoskeletal and Ageing Science, Institute of Life Course and Medical Sciences, University of Liverpool, West Derby Street, Liverpool, L7 8TX, United Kingdom; Department of Musculoskeletal and Ageing Science, Institute of Life Course and Medical Sciences, University of Liverpool, West Derby Street, Liverpool, L7 8TX, United Kingdom; Department of Clinical Biochemistry and Metabolic Medicine, Liverpool Clinical Laboratories, Royal Liverpool University Hospitals Trust, Mount Vernon Street, Liverpool, L7 8YE, United Kingdom; Institute of Systems, Molecular and Integrative Biology, University of Liverpool, Crown Street, Liverpool, L69 7BE, United Kingdom; Institute of Systems, Molecular and Integrative Biology, University of Liverpool, Crown Street, Liverpool, L69 7BE, United Kingdom; Liverpool Head and Neck Cancer Centre, University of Liverpool, West Derby Street, Liverpool, L7 8TX, United Kingdom; Department of Palliative Medicine, Clatterbridge Cancer Centre, Pembroke Place, Liverpool, L7 8YA, United Kingdom; Institute of Systems, Molecular and Integrative Biology, University of Liverpool, Crown Street, Liverpool, L69 7BE, United Kingdom; Department of Surgery, The Ottawa Hospital, Smyth Road, Ottawa, Ontario K1H 8L6, Canada; Department of Musculoskeletal and Ageing Science, Institute of Life Course and Medical Sciences, University of Liverpool, West Derby Street, Liverpool, L7 8TX, United Kingdom; Department of Clinical Biochemistry and Metabolic Medicine, Liverpool Clinical Laboratories, Royal Liverpool University Hospitals Trust, Mount Vernon Street, Liverpool, L7 8YE, United Kingdom; Department of Oncology and Metabolism, University of Sheffield, Beech Hill Road, Sheffield, S10 2RX, United Kingdom; Department of Musculoskeletal and Ageing Science, Institute of Life Course and Medical Sciences, University of Liverpool, West Derby Street, Liverpool, L7 8TX, United Kingdom

**Keywords:** osteoclasts, resorption, peptide fragments, collagen

## Abstract

Bone resorption involves dissolution of minerals and enzymatic degradation of bone matrix. The primary enzyme is cathepsin K but other proteases including matrix metalloproteinases are involved. Some cathepsin K cleavage products have been partially identified, including cross-linked telopeptides of type I collagen. Here, we aimed to characterize the entire complement of bone breakdown products resulting from osteoclast action under controlled conditions in vitro. We analyzed extracellular media from human osteoclasts cultured on dentin substrates, using untargeted liquid chromatography mass spectrometry. We discovered 22 breakdown products resulting from osteoclastic action. These products were peptide fragment sequences that mapped to various collagen proteins present in bone and dentin. Nine peptide fragments mapped exclusively to collagen I alpha-1 chain (COL1A1), the most abundant protein in bone. We subsequently detected 21 of the fragment products, initially observed in vitro, in human serum and/or urine. Consistent positive correlations were observed between the COL1A1-specific peptide fragments and established bone biochemical markers in serum and urine. Ten urine fragments and two serum fragments markedly increased (*p* < .05) following total hip arthroplasty, capturing the transient local peri-prosthetic osteolysis observed in these patients (serum, *n* = 86 patients; urine, *n* = 83 patients). Among these candidate osteolytic markers, four (two COL1A1-specific products) showed decreases from baseline (*p* < .05) in patients on denosumab (*n* = 10 patients). Additionally, two fragment peptides were higher (*p* < .05, fold change >2) in urine from patients with bone metastasis (24 out of 112) among a lung cancer cohort. The range of collagen peptide fragments we discovered as a direct result of osteoclast activity indicates a complexity of bone resorption pathways not previously known, extending beyond the known proteolytic cleavage events in bone collagen proteins. Monitoring biofluid concentrations of these novel bone markers has the potential to capture multiple pathways of bone resorption activity beyond the existing assays based on cathepsin K.

## Graphical Abstract

**Graphical Abstract:**
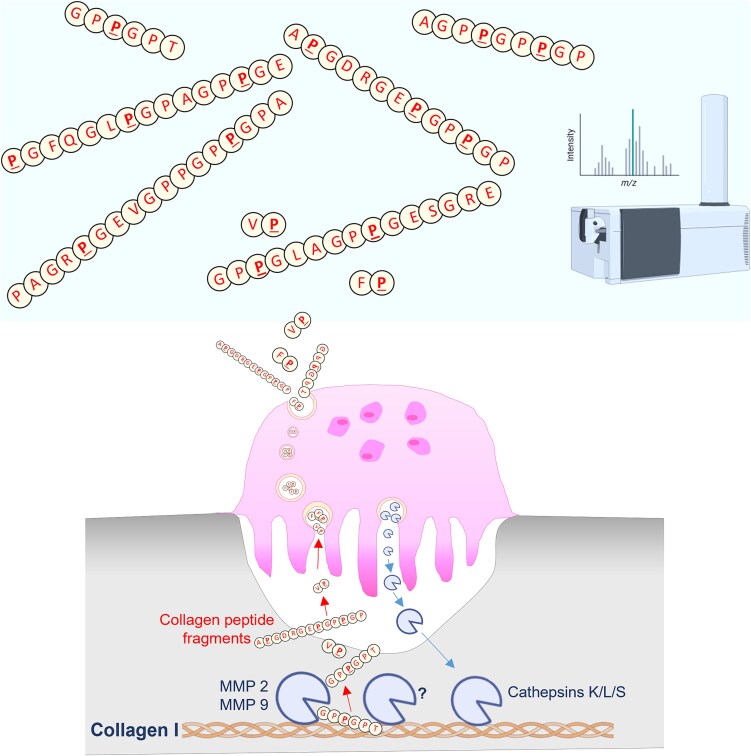

## Introduction

Osteoclasts are the principal cells that break down bone. Osteoclast action is fundamental to bone homeostasis, with an essential role in maintenance, repair, and remodeling of bone tissue. Osteoclasts resorb bone by producing various proteolytic enzymes and secreting hydrogen ions into the sealed extracellular compartment between the osteoclast and bone tissue matrix where resorption occurs.^1^ Bone resorption results in the breakdown of bone matrix proteins. The predominant protein in bone is type I collagen, which constitutes 90% of the organic component of bone matrix. Self-assembly of the 2 type I collagen chains, the alpha-1 (I) and alpha-2 (I) molecules, results in a protein structure comprising a triple helix with a coiled conformation.^2^

Specific degradation products of type I collagen produced during bone resorption by osteoclasts are well-established markers of bone turnover. Following release of these products from the tissue matrix, they enter the circulation and are then excreted in urine. Quantification of type I collagen breakdown products in biofluid compartments provides a noninvasive readout of bone resorption activity. This is the basis of several biochemical assays employed routinely in clinical management of bone disorders. The existing biochemical bone resorption markers produced by the enzyme cathepsin K do not represent the entirety of bone breakdown pathways by osteoclasts. Multiple studies have shown that even in healthy subjects, human serum and urine contain a range of peptide products derived from collagens, particularly collagen I alpha-1 chain (COL1A1).^3–8^ The variety of urine collagen fragments indicates that the protease cleavage sites in type I collagen I are extensive and not limited to the well-characterized cathepsin K cleavage sites.^5^ It is likely that some type I collagen fragments are the products of other proteolytic enzymes. For example, a group of non-cysteine protease enzymes expressed in bone and known to act on collagen is the matrix metalloproteinases (MMPs).^9^

A recent study demonstrated the potential of an approach based on proteolysis of bone under controlled conditions in the laboratory, combined with high-resolution mass spectrometry (MS), for characterizing the global breakdown pathways of collagens.^10^ Digestion of human type I collagen with purified cathepsin K resulted in the production of more than 100 peptide products originating from each of the COL1A1 and COL1A2 proteins. These products included peptides from known cathepsin K cleavage sites, in addition to a range of previously uncharacterized peptides. This indicates new cleavage sites not only limited to the cross-linking telopeptide domains but also including the helical portion of these proteins. The degree to which these products reflect osteoclastic resorption as it occurs in vivo, or if they are present in human blood or urine, is not known.

To provide a more physiologically representative view of osteoclast resorption by proteases not limited to cathepsin K, we studied the resorption products generated by activated osteoclasts cultured on dentin substrates. The organic matrix component of dentin, like bone, is comprised predominantly of type I collagen.^11^ We then established the extent to which these identified breakdown products were observed in human serum and urine. Understanding the complexity of bone resorption pathways is a fundamental knowledge in basic bone biology and has potential to inform new approaches for monitoring and treatment of disorders associated with dysregulation of bone remodeling.

## Materials and methods

### Chemicals and reagents

Human osteoclast precursors, culture medium, and medium supplements were from Lonza. Base medium was OCP basal medium. Supplements and growth factors were from the OCP growth medium SingleQuots kit. Final growth medium composition was 10% fetal bovine serum, 2 mM L-glutamine, 100 units/mL penicillin/streptomycin, 33 ng/mL macrophage colony-stimulating factor (M-CSF), and 66 ng/mL receptor activator of nuclear factor kappa-beta ligand (RANKL).

For liquid chromatography mass spectrometry (LC/MS) analysis, deionized water was purified in-house by DIRECT-Q 3UV water purification system (Millipore). Methanol, isopropanol (Sigma-Aldrich), formic acid (Biosolve), and ammonium formate (Fisher Scientific) were LC/MS grade. Reference mass correction solution was prepared in 95:5 methanol:water containing 5 mmol/L purine (CAS No. 120-73-0) and 2.5 mmol/L hexakis (1H, 1H, 3H-tetrafluoropropoxy)phosphazine (HP-0921, CAS No. 58943-98-9) (Agilent).

### In vitro dentin resorption assay

Dentin disks were prepared from hippo and walrus tusks. Disks were placed into wells of a 96-well plate. Human osteoclast precursor cells were cultured and differentiated into osteoclasts according to manufacturer guidelines. Osteoclast precursors were seeded into 96-well culture plates onto hippo dentin, walrus dentin, or plastic at a density of 10 000 precursor cells per well in 100 μL growth medium containing M-CSF and RANKL for osteoclast generation. At day 7 and 14 of culture, 50 μL (50%) and 100 μL (100%) of medium, respectively, from each well was replaced with fresh growth medium. After 19 d, culture medium was harvested and stored at −80 °C until analysis. Dentin wafers were fixed in phosphate buffered saline containing 10% formalin, stained with toluidine blue, and examined using reflected light microscopy to quantify resorption lacunae. Total resorption area for each dentin disk was quantified using an Olympus BH2 microscope (×10 objective) fitted with a source of incident light and a drawing tube to enable projection of a grid over the visual field. Resorption area was determined with aid of the projected grid using the point counting technique previously described.^12^ Non-resorptive control conditions were osteoclast precursors cultured onto plastic disks (+RANKL/−dentin; *N* = 6) or without RANKL (−RANKL/+dentin; *N* = 6, 3 walrus disks, 3 dentin disks), or onto plastic and without RANKL (−RANKL/−dentin; *N* = 3). Additional +RANKL/+dentin cultures were treated with 10 fM-100 μM zoledronic acid (6 concentrations, *N* = 3 per concentration) at day 2, to examine potential effects of anti-resorptive treatment.

### LC/MS analysis

Extracellular media samples were thawed at room temperature then diluted 1:3 media: deionized water (v/v) in individual 1.5 mL microcentrifuge tubes. Sample tubes were vortexed for 10 s then centrifuged at 1500 × *g* for 5 min. Fifty microliter of supernatant was transferred to a 150 μL 96-well plate (Agilent) for analysis. Pooled samples were created for quality control (QC) purposes for each treatment condition in addition to an overall QC by pooling equal volumes of individual samples. Pooled samples were prepared in an identical way to individual samples.

Human serum and urine samples (see “Human metabolomic data” section below) were stored at −80 °C until preparation. Urine samples were analyzed following 1:3 (v/v) dilution urine: deionized water in 700 μL 96-well polypropylene sample collection plates (Waters). Serum samples were analyzed following deproteinization with ice-cold methanol. Two-hundred microliters of serum was added to 600 μL methanol contained in 1.5 mL microcentrifuge tubes on ice, then vortexed for 10 s and centrifuged at 16 000 × *g*, 4 °C to precipitate protein. One-hundred microliters of supernatant extract was transferred to a 700 μL 96-well polypropylene sample collection plate (Waters) then methanol extracts were dried at room temperature under compressed air (<1 h). Dried extracts were stored at −80 °C until analysis, at which point they were reconstituted in 200 μL deionized water and agitated on a plate shaker (MTS 2/4 m IKA, Germany) at 800 rpm for 10 min before analysis. Pooled samples were created for human samples in each study for QC purposes, as described above.

Sample analysis was performed using a published liquid chromatography quadrupole time-of-flight mass spectrometry (LC-QTOF-MS) acquisition method, which employed a 1290 Infinity II HPLC coupled to a 6550 QTOF-MS equipped with dual AJS electrospray ionization source (Agilent).^13^ Sample injection volume was 10 μL for culture medium, 1 μL for urine, and 2 μL for serum. Reversed-phase LC was performed on an Atlantis dC18 column (3 × 100 mm, 3 μm, Waters) maintained at 60 °C with mobile phase flow rate 0.4 mL/min. Mobile phase composition was (A) water and (B) methanol, both with 5 mmol/L ammonium formate and 0.1% formic acid. The elution gradient started at 5% B 0-1 min and increased linearly to 100% B by 12 min, held at 100% B until 14 min, then at 5% B for a further 5 min. MS data acquisition was performed in positive ionization polarity with mass range 50-1700 in 2 GHz mode with acquisition rate at 3 spectra/s. A reference mass correction solution was continually infused at a flow rate of 0.5 mL/min via an external isocratic pump (Agilent) for constant mass correction (see preparation of reference mass correction solution). Capillary and fragmentor voltages were 400 and 380 V, respectively. Desolvation gas temperature was 200 °C with flow rate at 15 L/min. Sheath gas temperature was 300 °C with flow rate at 12 L/min, and nebulizer pressure was 40 psi and nozzle voltage 1000 V. Reference ions monitored were: purine (*m/z* 121.0509) and HP-0921 (*m/z* 922.0098).

The LC/MS analytical sequence in each study was designed following published guidance.^14^ Injection order of individual samples was randomized computationally using Microsoft Excel. Pooled QC samples were interspersed throughout each analytical sequence, every 10th injection. Samples from each study were analyzed in one analytical batch except for the total hip arthroplasty study; sample preparation and analysis split over two representative batches.

Compound fragmentation spectra for resulting analytes of interest from the in vitro study were obtained from MS2 analysis of media samples with accurate mass precursor ion targets detailed in Table 1; no more than 6 compounds targets per injection. Multiple fixed collision energies were applied; 10, 20, and 40 eV. Acquisition rates were 6 spectra/s in MS1 and 4 spectra/s in MS2.

### Human metabolomic data

Human serum and urine untargeted metabolomic datasets were acquired under identical analytical conditions to those described above for in vitro experiment samples.

Serum and urine data were acquired on samples obtained from 2 clinical trials conducted in patients undergoing total hip arthroplasty. One study compared 2 femoral prosthesis designs in patients undergoing total hip arthroplasty for hip osteoarthritis at The Ottowa Hospital, Ottowa, Canada between 2013 and 2017.^15^ The conventional prosthesis was associated with a smaller reduction in peri-prosthetic bone mineral density loss compared with a Tri-Lock Bone Preservation Stem femoral prosthesis. No statistically significant difference was observed between the two prosthesis designs in the profiles of established biochemical bone turnover markers serum CTX and P1NP. Serum and urine (total serum samples, *n* = 334; total urine samples, *n* = 374) were collected at five time points across the study: baseline (pre-surgery) then at 3, 6, 12, and 24 mo post-surgery. Serum samples were from 86 patients (40 male, 46 female, mean age [±SD] 59 ± 11 yr) and urine samples were from 83 patients (39 male, 44 female, mean age 59 ± 11 yr). The second study was a phase 2 randomized, double-blind, placebo-controlled trial carried out at Sheffield Teaching Hospitals, Sheffield, UK between 2012 and 2018 to investigate the effect of the monoclonal antibody denosumab on osteolytic activity in patients listed for revision total hip arthroplasty surgery. The denosumab group showed 83% fewer osteoclasts at the osteolysis membrane-bone interface compared with the placebo group.^16^ Serum and urine (total serum samples, *n* = 89; total urine samples, *n* = 94) were collected from patients immediately before a single subcutaneous injection of 60 mg denosumab or placebo control (study visit 2), then again at 4 (study visit 3) and 8 wk (study visit 4) on treatment. Serum samples were from 21 patients: 10 in the denosumab group (6 male, 4 female, mean age 72 ± 12 yr) and 11 in the placebo group (9 male, 2 female, mean age 71 ± 9 yr). Urine samples were from 22 patients: 10 in the denosumab group (6 male, 4 female, mean age 72 ± 12 yr) and 12 in the placebo group (10 male, 2 female, mean age 72 ± 9 yr). Serum and urine from both total hip arthroplasty sample sets were collected following complete overnight fast. In the UK study, patients had not received bisphosphonates (no oral bisphosphonate therapy within 12 mo of the study, no intravenous bisphosphonates within 5 yr of the study), and in the Canadian study, patients had no past or present use of drugs known to affect bone metabolism.

Additional urine metabolomic data were from a study investigating the metabolic changes that occur during the dying process in patients with incurable lung cancer (patients *n* = 112).^17^ This dataset is available at the NIH Common Fund’s National Metabolomics Data Repository website, the Metabolomics Workbench (https://www.metabolomicsworkbench.org/); Project ID PR001322, directly accessible via Project DOI: 10.21228/M8TT44. Serial urine samples were collected from patients at various hospitals/hospices in northwest England between 2016 and 2018 at various time points leading up to death (>12 to <1 wk before death); total samples *n* = 233. Among the 112 patients in the urine study, 24 patients (12 male, 12 female, mean age 69 ± 9 yr) had bone metastasis confirmed by CT, PET, or bone scintigraphy; the other 88 patients (55 male, 33 female, mean age 70 ± 10 yr) had lung cancer but without known bone metastasis.

Research on these human datasets was performed under the following ethical approvals. Total hip arthroplasty datasets: approval by Ottawa Hospital Institutional Review Board (OHSN-REB 2010913-01H) and registered with clinicaltrials.gov (NCT01558752); for the denosumab trial, approval by National Research Ethics Service Committee Yorkshire and the Humber-Leeds West (REC reference 11/YH/0252) and registered with the EU Clinical Trials Register (EudraCT 2011-000541-20). Biology of dying urine dataset: approval provided by North Wales (West) Research Ethics Committee (REC reference 15/WA/0464).

### Data processing and statistical analysis

Raw LC/MS data were mined for compound features in Masshunter Profinder (build 10.00, Agilent). Data from in vitro media samples were mined by an untargeted approach using the batch recursive (small molecules/peptides) feature extraction function. Untargeted feature extraction parameters: peak height >5000 counts, allowed ion species +H, +Na, +K, and +NH_4_, alignment retention time (RT) tolerance ±0.3 min, alignment mass tolerance ±20 ppm, molecular feature extraction score >70, and Agile 2 peak integration method. Compounds not detected in all individual samples across at least one treatment condition were removed from the analysis.

Human metabolomic data were mined for the oligopeptide features identified from the in vitro experiment using the targeted feature extraction function in Profinder. Feature extraction parameters were accurate mass match window ±5 ppm and RT window ±0.3 min. Allowed ion species were + H, +Na, +K and + NH_4_. “Find by formula” filters were: score >60 in at least 60% of samples from at least one sample group.

Extracted peak area intensity data were exported in .csv file format and imported into Mass Profiler Professional (MPP; build 15.1, Agilent). In MPP, all data were log_2_ transformed and pareto scaled. Urine data from the total hip arthroplasty studies were normalized to total peak area, ascertained from untargeted metabolite feature extraction (Mass Profiler; version B.07.02, build 123.0, Agilent). Urine data from the biology of dying study were normalized to urine creatinine concentration. QC of extracted features was performed on all datasets as previously described, based on reproducibility of signals across replicate injections of each pooled sample; features present in 100% of injections for at least one pooled sample, and with coefficient of variation (CV) <30% across replicate injections were retained in the analysis.^13^ Statistical comparisons between in vitro experiment groups were performed in IBM SPSS (version 31) by Kruskal-Wallis test and post-hoc analysis by Dunn’s test, both with Benjamini-Hochberg false-discovery rate (FDR) multiple testing correction. Potential longitudinal differences in the abundance of urine and serum compounds in the total hip arthroplasty datasets were assessed in MPP by repeated-measures ANOVA and post-hoc analysis by pairwise repeated measures *t*-tests (versus baseline visit), both with Benjamini-Hochberg FDR adjustment. In the lung cancer urine dataset, volcano plot analysis combining fold-change (FC) and T-test analyses were performed in MetaboAnalyst (version 6.0)^18^ to examine potential differences in urine compounds between patients with versus without bone metastasis.

### Compound structure identification

Compound identification was performed using multiple chemical properties; accurate mass and MS2 fragmentation spectra of extracted features, in addition to the known amino acid sequences of the major extracellular matrix proteins present in dentin. Extracellular proteins mapped to were collagens 1, 2, 3, and 5; COL1A1, COL1A2, COL2A1, COL3A1, COL5A1, and COL5A2. Reference amino acid sequences for these proteins, with hydroxyproline and hydroxylysine post-translational modifications, were obtained from the NCBI protein database (National Library of Medicine; National Institutes of Health; U.S. Department of Health and Human Services) with the following accession numbers: NP_000079.2 (COL1A1), NP_000080.2 (COL1A2), NP_001835.3 (COL2A1), NP_000081.2 (COL3A1), NP_000084.3 (COL5A1), NP_000384.2 (COL5A2). The theoretical monoisotopic neutral accurate masses of all possible oligopeptide chain sequences 1-20 amino acids long for these proteins were mapped out in Microsoft Excel. Matching of calculated neutral masses of observed features was performed against these sequence mass databases generated for each candidate protein (mass match window ±5 ppm).

## Results

### Resorption of dentin disks in vitro

**Figure 1:**
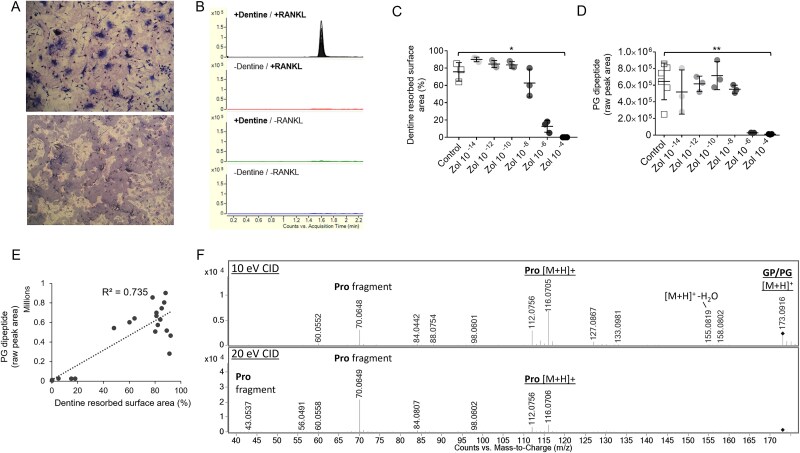
Observation of bone resorption in the dentin slice assay and identification of oligopeptide biochemical markers of bone matrix breakdown. (A, upper panel) Transmitted light photomicrograph showing osteoclasts resorbing dentin substrate. (Lower panel) reflected light photomicrograph showing resorption trails. (B) Representative chromatogram of an oligopeptide bone resorption marker from extracellular media sampled from the dentin slice assay. Note the exclusive presence of signal in cultures of RANKL-activated osteoclast precursors grown on dentin but no signal detected in any of the control conditions. Traces from individual samples are overlaid within each treatment group. (C, D) Inhibition of dentin resorption and peptide fragment release into extracellular media with zoledronic acid treatment 10^−14^ M (10 fM)-10^−4^ M (100 μM) in activated osteoclast cultures. Dot plots show inhibition of osteoclast resorption between 10^−8^ and 10^−6^ M zoledronic acid concentrations, indicated by sharp reduction in the total resorbed surface area of dentin disks (C) and abundance of oligopeptide fragments detected in media (D). ^*^p < .05; ^**^p < .01. (E) Positive correlation between oligopeptide fragment signal and total resorbed surface area of dentin slices across zoledronic acid treated cultures 10^−14^ M (10 fM)-10^−4^ M (100 μM). (F) Example MS/MS fragmentation spectra for peptide fragment at multiple collision energies, showing the intact molecular [M + H]^+^ cation and MS peaks representing constituent substructures, including the individual amino acid proline. The representative peptide fragment product shown in sections (B-F) is the dipeptide comprised of proline and glycine. Abbreviation: CID = collision-induced dissociation.

Extensive resorption of dentin disks was observed in cell cultures grown on dentin substrate with activation of osteoclast activity by RANKL supplementation in growth medium (+dentin/+RANKL). In these cultures, resorption pits covered >60% of the dentin surface area (Figure 1A). No difference was observed in extent of resorption between hippo and walrus dentin. Resorption of dentin substrates was not observed in control cultures without RANKL supplementation.

### Oligopeptide markers of in vitro dentin resorption in extracellular media

Untargeted feature extraction of LC/MS data acquired from extracellular media resulted in 153 positively charged ion features across all samples. QC filtering resulted in 148 features present in all replicate injections for at least one pooled group sample; 147 of these features passed additional variability filtering, with CV <25% across replicate injections of each pooled sample. Thirty-one features had increased abundance (*p* < .05, fold change >2) in resorption-positive +dentin/+RANKL osteoclast cultures compared with all control cultures. Twenty-two of these features were identified as potential resorption markers following additional QC checks on raw data for visualization of feature integration quality and removal of duplicate extracted features (Table 1). Among these chemical entities, 21 compounds were exclusively present in +dentin/+RANKL cultures (Figure 1B and F), except for glycyl-prolyl-hydroxyproline, which was also detected in control cultures at lower abundance; raw peak area mean (±SD) = 364 326 (±73 573) in +dentin/+RANKL, mean (±SD) = 45 755 (±15 310) across control cultures (Figure S1).

The abundance of 21 out of 22 resorption compounds showed a clear effect of zoledronic acid treatment in +dentin/+RANKL cultures; *p* < .05. There was strong concordance between the profiles of these compounds, with a consistent sharp decline at zoledronic acid concentration ≥1 μM (Figure 1C and D). Post-hoc analysis confirmed this trend, with significant differences (*p* < .05 or <0.01) between the 100 μM zoledronic acid groups compared with untreated control for 21 out of 21 zoledronic acid responsive compounds. Significantly lower signal (*p* < .05) was also observed at 1 μM zoledronic acid, versus control, for 11 of these 21 compounds. Strong positive correlations were observed between peptide fragment abundance and the quantified resorption area of dentin disks across the zoledronic acid treatment groups (mean ± SD, *R*^2^ = 0.72 ± 0.11; Figure 1E). Inhibition with zoledronic acid treatment was not observed for the compound identified as the dipeptide His-Hyp (or Hyp-His; *m/z* 251.1142) despite not being detected in the −dentin/+RANKL, +dentin/−RANKL, or −dentin/−RANKL controls (Figure S2).

### Chemical identification and mapping oligopeptide fragments to matrix proteins

**Figure 2:**
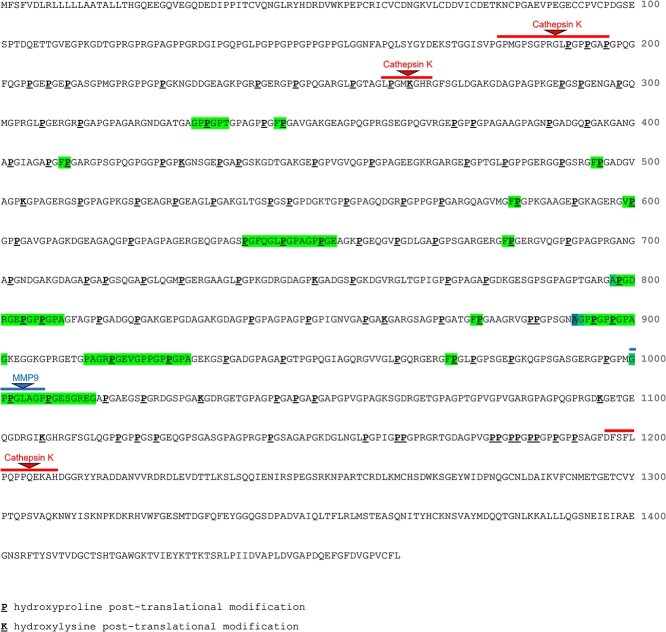
Sequence positions of peptide fragments (highlighted) that mapped exclusively to the human COL1A1 protein. Among these fragment sequences, 1/9 overlapped the position of a reported protease cleavage sites in COL1A1^19^ (MMP9). The reported cleavage sites of cathepsin K are shown for reference.

With exception of one compound (compound #13, Table 1), all compounds mapped to one or more oligopeptide amino acid sequence present in the extracellular matrix proteins COL1A1, COL1A2, COL2A1, COL3A1, COL5A1, or COL5A2 (Table 1, Table S1). Eleven of the 22 compounds were identified as various di-peptides and mapped to multiple sequences across the proteins examined, except for three dipeptides that were unique to COL1A1. Nine of the 22 compounds mapped exclusively to the COL1A1 protein. These COL1A1-specific oligopeptides ranged from 2 (Val-Hyp and Phe-Hyp) to 17 amino acids. The five highest MW products were all COL1A1 specific and mapped uniquely to single sequences within this protein (Figure 2). These COL1A1-specific compounds were doubly charged and contained a minimum of one Gly-Pro-Hyp sequence. Four compounds were identified as products of dipeptides minus the accurate mass of H_2_O. These molecules are unlikely to be technical artifacts caused by MS in-source fragmentation, as the chromatographic RT was different to their derivative complete dipeptides where these were observed.

### Comparison of COL1A1 fragment peptide sequence locations to known COL1A1 cleavage sites

Known proteolytic cleavage sites in the COL1A1 protein were investigated to identify candidate collagenolytic enzymes with the potential to produce the COL1A1 peptide fragments discovered. Using the MEROPS database of proteolytic enzymes,^19^ COL1A1 was selected as a substrate protein and all reported cleavage sites across all reported COL1A1-targeting peptidases were identified. These peptidases included cathepsins D, L, K, and S and the MMPs. All COL1A1 cleavage sites reported from any form of evidence in this database were considered. All our identified COL1A1 peptide fragments greater than 3 amino acid residues were considered in this analysis, except for the di-peptides Phe-Hyp and Val-Hyp which were also considered. These fragments, and particularly the longer sequences, were generally more specific to COL1A1.

Table 1 shows the predicted proteases associated with each peptide fragment based on overlap between their COL1A1 mapping positions and all known COL1A1 protease cleavage sites provided in MEROPS. The COL1A1 protein sequence positions of four of our peptide fragments overlapped with a known COL1A1 proteolytic cleavage site. These known cleavage sites are targeted by the collagenolytic enzymes cathepsins K, L, and S, MMP2 and MMP9. Figure 2 shows the COL1A1 sequence positions of the 9 peptide fragments that mapped exclusively to COL1A1. Only 1 out of 9 COL1A1-specific peptides overlapped with a known cleavage site; a 15-amino acid residue peptide fragment which overlapped with one cleavage site reported for MMP9 (compound #20; see Table 1). None of our other observed peptide fragments greater than 5 amino acid residues overlapped a known COL1A1 cleavage site.

**Table 1:** Summary of bone resorption products observed from LC/MS analysis.

Compound ID	Proposed compound identification	Amino acid sequence	Proposed empirical formula	Human biofluids detected in	Possible originating proteins	Predicted proteases
**1**	Gly-Pro/Pro-Gly	GP/PG	C_7_ H_12_ N_2_ O_3_	Serum, urine	COL1A1, COL1A2, COL2A1, COL3A1 COL5A1, COL5A2	
**2**	Val-Gly/Gly-Val	VG/GV	C_7_ H_14_ N_2_ O_3_	Serum, urine	COL1A1, COL1A2, COL2A1, COL3A1 COL5A1, COL5A2	
**3**	Hyp-Ala/Ala-Hyp (-H_2_O)	*P*A/A*P*	C_8_ H_12_ N_2_ O_3_	Serum, urine	COL1A1, COL1A2, COL2A1, COL3A1, COL5A2	
**4**	Pro-Ser/Ser-Pro/Ala-Hyp	PS/SP/A*P*	C_8_ H_14_ N_2_ O_4_	Serum, urine	COL1A1, COL1A2, COL2A1, COL3A1 COL5A1, COL5A2	
**5**	Leu-Hyp/Hyp-Leu Ile-Hyp/Hyp-Ile (-H_2_O)	L*P*/*P*L/I*P*/*P*I	C_11_ H_18_ N_2_ O_3_	Urine	COL1A1, COL2A1 COL3A1, COL5A1	
**6**	Val-Hyp	V*P*	C_10_ H_18_ N_2_ O_4_	Urine	COL1A1	
**7**	Leu-Hyp/Hyp-Leu Ile-Hyp/Hyp-Ile	L*P*/*P*L/I*P*/*P*I	C_11_ H_20_ N_2_ O_4_	Serum, urine	COL1A1, COL2A1 COL3A1, COL5A1, COL5A2	
**8**	Hyp-His/His-Hyp (-H_2_O)	*P*H/H*P*	C_11_ H_14_ N_4_ O_3_	Urine	COL3A1, COL5A1	
**9**	Phe-Hyp (-H_2_O)	F*P*	C_14_ H_16_ N_2_ O_3_	Serum	COL1A1	
**10**	Phe-Hyp	F*P*	C_14_ H_18_ N_2_ O_4_	Serum, urine	COL1A1	
**11**	Gly-Pro-Hyp	GP*P*^a^	C_12_ H_19_ N_3_ O_5_	Serum, urine	COL1A1, COL1A2, COL2A1	Cathepsins K/L/S, MMP2, MMP9
**12**	Arg-Hyp	R*P*	C_11_ H_21_ N_5_ O_4_	Urine	COL1A1, COL1A2,	
**13**	Unknown (MW: 307)	-	-	Urine		
**14**	Gly-Pro-Hyp-Gly	GP*P*G^a^	C_14_ H_22_ N_4_ O_6_	Serum, urine	COL1A1, COL1A2, COL2A1	Cathepsins K/L/S, MMP2, MMP9
**15**	Multiple candidates	PGP*P*G/GP*P*GP/PGGP*P*/*P*GPPG^a^	C_19_ H_29_ N_5_ O_7_	Urine	COL1A1, COL1A2, COL2A1	Cathepsins K/S, MMP2, MMP9
**16**	Gly-Pro-Hyp-Gly-Pro-Thr	GP*P*GPT	C_23_ H_36_ N_6_ O_9_		COL1A1	
**17**	Multiple candidates	P*P*GP*P*GP/GP*P*GP*P*G	C_26_ H_39_ N_7_ O_10_	Urine	COL1A1, COL1A2	
**18**	Multiple candidates^c^	AGP*P*GP*P*GP/GP*P*GP*P*GPA/P*P*GP*P*GPAG	C_34_ H_51_ N_9_ O_12_	Urine	COL1A1	
**19**	Multiple candidates^c^	A*P*GDRGE*P*GP*P*GP/*P*GDRGE*P*GP*P*GPA^b^	C_51_ H_78_ N_16_ O_21_	Serum, urine	COL1A1	
**20**	Multiple candidates^c^	GP*P*GLAGP*P*GESGRE/ P*P*GLAGP*P*GESGREG^a^	C_58_ H_92_ N_18_ O_23_	Urine	COL1A1	MMP9
**21**	Hyp-Gly-Phe-Gln-Gly-Leu-Hyp-Gly-Pro-Ala-Gly-Pro-Hyp-Gly-Glu	*P*GFQGL*P*GPAGP*P*GE^a^	C_63_ H_92_ N_16_ O_22_	Urine	COL1A1	
**22**	Pro-Ala-Gly-Arg-Hyp-Gly-Glu-Val-Gly-Pro-Pro-Gly-Pro-Hyp-Gly-Pro-Ala	PAGR*P*GEVGPPGP*P*GPA	C_67_ H_104_ N_20_ O_22_	Serum, urine	COL1A1	

Proposed identifications are based on mapping of observed peptide fragments against all possible oligopeptide products obtained from amino acid sequences for mature collagen proteins present in dentin. Predicted proteases are those proposed to produce the observed oligopeptides based on overlap between our markers and known protease cleavage sites in the COL1A1 protein; octapeptide cleavage sites obtained for cathepsin proteases and matrix metalloproteinases (MMPs) from the MEROPS^19^ database. MW (neutral monoisotopic mass). Abbreviation: *P* = hydroxyproline.

^a^Compound overlaps sequence position of a previously-reported urine COL1A1 peptide fragment^5^ (for fragments of >3 amino acid residues). ^b^Compound exactly reproduces a previously-reported urine COL1A1 peptide fragment.^5^

^c^Multiple possible positional isomers originating from the same singular amino acid sequence in COL1A1 (see Figure 2).

### Novel oligopeptide markers in human serum and urine

**Figure 3:**
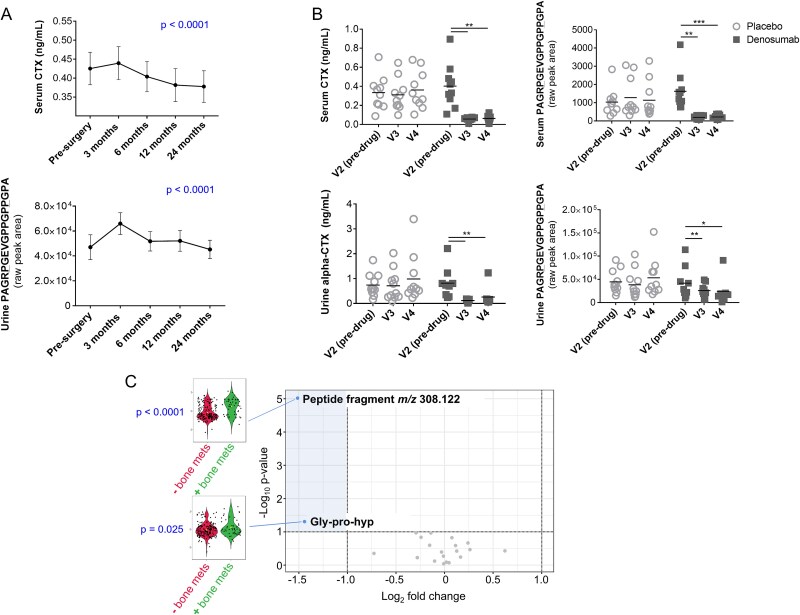
Oligopeptide products as potential markers of bone resorption and metastatic bone disease in humans. (A) Longitudinal abundance profiles of biochemical analytes from a total hip arthroplasty patient cohort.^15^ Serum and urine samples were taken at baseline (pre-surgery) then at 3, 6, 12, and 24 mo post-surgery. Analytes are serum CTX (data from Slullitell et al.^15^), and the largest COL1A1-specific peptide fragment identified in this study (compound 22, Table 1). Plots show data from all samples (serum, *n* = 334; urine, *n* = 374). *p*-values are from repeated measures one-way ANOVA on patients (serum, *n* = 49; urine, *n* = 52) with samples available for all time points, adjusted to false-discovery rate by Benjamini-Hochberg method. (B) Serum and urine biochemical abundance profiles in patients on denosumab/placebo at 4 (visit 3) and 8 wk (visit 4) on treatment versus baseline (visit 2). Compound 22 shown as an example peptide fragment with decreased abundance from baseline in patients on denosumab in serum and urine (^*^p < .05, ^**^p < .01, ^***^p < .001; Benjamini-Hochberg false discovery rate adjusted). This trend was in line with the published data on established bone markers in this cohort; decreases in serum and urine CTX on denosumab with no decrease in the placebo group.^16^  *p*-Values are from repeated measures ANOVA on patients (serum data, denosumab *n* = 8 patients, placebo *n* = 11 patients; urine data, denosumab *n* = 7 patients, placebo *n* = 9 patients) with samples available for all three time points. Dot plots show data from all samples analyzed (serum, *n* = 58; urine, *n* = 58). (C) Volcano plot highlighting the two oligopeptide compounds higher in urine from patients with bone metastasis among the lung cancer cohort (markers in shaded region indicating *p* < .05 and fold change >2 between patients with bone metastasis versus controls). These compounds were gly-pro-hyp and an unidentified compound with m/z 308.122 (compound #13, Table 1). All remaining urinary oligopeptides detected showed no significant difference (*p* > .05 and fold change <2) between patient groups (gray dots). *N* = 24 patients with lung cancer and bone metastasis (mets), *n* = 88 patients with lung cancer but without known bone metastasis.^17^

Serum and urine untargeted metabolomic datasets acquired in our laboratory were mined for the aforementioned oligopeptide products. The total hip arthroplasty datasets provided the opportunity to assess longitudinal profiles of the oligopeptides in serum and urine among patient cohorts with characteristic alteration in established biochemical bone turnover markers resulting from (1) acute post-surgical periprosthetic osteolysis^15^ (Figure 3A) and (2) attenuation of local osteolytic lesions with denosumab^16^ (Figure 3B). We also mined urine data from a study designed to characterize the urine metabolite changes occurring in terminally ill lung cancer patients at various time points leading up to death.^17^ Among this cohort, 24 out of 112 patients had known bone metastasis, providing the opportunity to examine oligopeptide profiles in this bone disorder.

Twenty-one of the 22 peptide fragments, we discovered from in vitro experiments, were detected in human serum and/or urine; 11 fragments in serum, 20 fragments in urine (Table 1). Signals for these molecules were reproducible, with 100% frequency and peak area CV <30% across replicate injections of pooled samples in each analytical run.

In the Canadian hip arthroplasty dataset, a subset of the molecules increased at the visit immediately following total hip replacement (3 mo post-surgery), in line with the previously reported increases in established bone markers serum CTX and P1NP at this visit.^15^ A total of 3 out of 8 serum oligopeptides and 10 out of 20 urine oligopeptides showed longitudinal differences in abundance over the 2-yr study period (*p* < .05) (Figure 3A, Figures S3 and S4). Two of these three serum oligopeptides and all 10 of these urine oligopeptides showed a clear increase at 3 mo post-surgery, as with serum CTX and P1NP, compared with baseline. Among the statistically non-significant urine oligopeptides, 6 out of 10 showed this same general trend.

In the denosumab dataset, 4 out of 11 serum oligopeptides detected showed decreases at 4 (visit 3) and/or 8 wk (visit 4) on denosumab versus baseline (*p* < .05). No statistically significant difference from baseline was observed for these molecules in the placebo group (Figure 3B, Figure S5). In serum, the most marked decrease from baseline in denosumab-treated patients was for the largest, COL1A1-specific, peptide fragment identified in this analysis (compound 22, Table 1); mean fold change from baseline = 7.5 at 4 wk (*p* < .01) and 7.2 at 8 wk (*p* < .001). In urine, this compound also decreased in denosumab-treated patients; mean fold change from baseline = 1.9 at 4 wk (*p* < .01) and 2.3 at 8 wk (*p* = .036) (Figure 3B). No statistically significant differences were observed from baseline for the other urine oligopeptides in denosumab or placebo groups.

**Figure 4:**
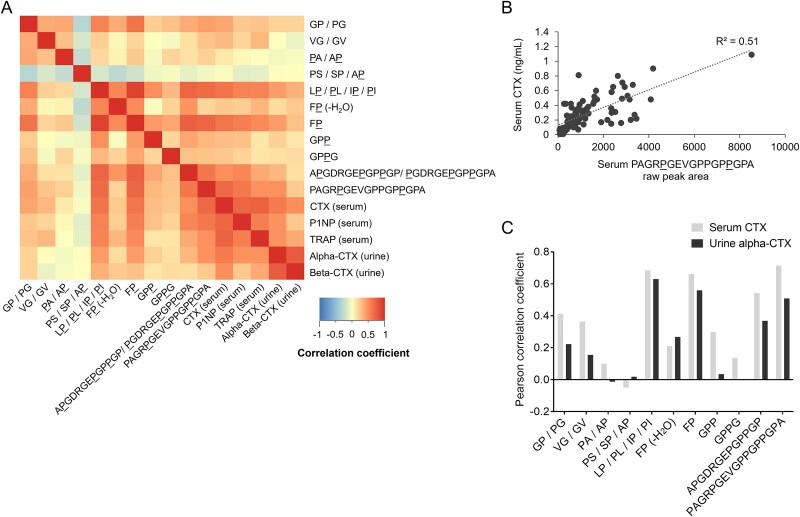
Correlation between serum collagen peptide fragments and established bone resorption markers. (A) Heatmap from Pearson correlation analysis between all collagen peptide fragments identified in serum and established biochemical bone markers. (B) Scatter plot showing correlation between serum CTX and the largest COL1A1-specific peptide identified in this analysis (compound 22, Table 1). (C) Summary of Pearson correlation coefficients between the collagen peptide fragments detected in serum and serum CTX and urine alpha-CTX. All data are from the denosumab clinical trial sample set (*n* = 89 samples).^16^

**Figure 5:**
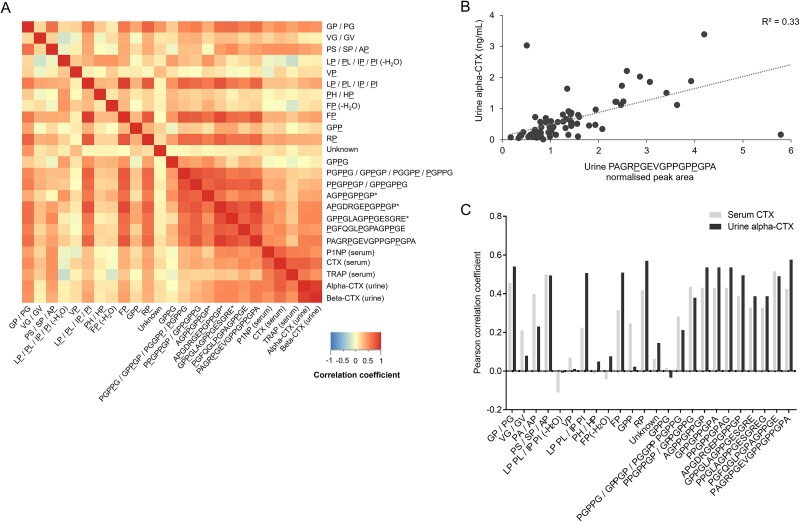
Correlation between urine collagen peptide fragments and established bone resorption markers. (A) Heatmap from Pearson correlation analysis between all collagen peptide fragments identified in urine and established biochemical bone markers. (B) Scatter plot showing correlation between urine alpha-CTX and the largest COL1A1-specific peptide identified in this analysis (compound 22, Table 1). (C) Summary of Pearson correlation coefficients between the collagen peptide fragments detected in urine and serum CTX and urine alpha-CTX. All data are from the denosumab clinical trial sample set (*n* = 94 samples).^16^

Correlation analysis was performed on the denosumab dataset (data from all visits), as data from established bone biochemical markers in serum and urine were available for this study. Pearson analysis showed positive correlations between 8 out of 11 serum peptide fragments and serum CTX (*p* < .05, correlation coefficient range = 0.21-0.71) (Figure 4). Out of the 21 urine peptide fragments detected, 12 compounds correlated (*p* < .05) with urine alpha-CTX (correlation coefficient range = 0.23-0.58) and 13 compounds correlated with beta-CTX (*p* < .05, correlation coefficient range = 0.27-0.61) (Figure 5). The largest, COL1A1-specific peptide fragments generally showed the most consistent correlations with established bone markers (Figures 4A and C and 5A and C); also including TRAP and P1NP in addition to serum and urine CTX. Table 2 summarizes the data from correlation analysis between all peptide fragments and clinical bone biochemical markers investigated.

**Table 2:** Summary of Pearson correlation analysis of collagen peptide fragments against established clinical biochemical bone markers.

Compound ID	Peptide fragment	Serum CTX		Serum TRAP		Serum P1NP		Urine alpha-CTX		Urine beta-CTX	
		Serum	Urine	Serum	Urine	Serum	Urine	Serum	Urine	Serum	Urine
**1**	GP/PG	0.413^b^	0.455^b^	0.262^a^	0.390^b^	0.310^b^	0.514^b^	0.222	0.540^b^	0.256^a^	0.493^b^
**2**	VG/GV	0.364^b^	0.210^a^	0.241^a^	0.083	0.161	0.143	0.155	0.079	0.106	0.070
**3**	PA/AP	0.099	0.399^b^	−0.04	0.222^a^	0.142	0.329^b^	−0.013	0.230^a^	−0.015	0.282^a^
**4**	PS/SP/A*P*	−0.049	0.498^b^	−0.125	0.483^b^	−0.088	0.422^b^	0.018	0.494^b^	−0.022	0.408^b^
**5**	L*P*/*P*L/I*P*/*P*I (-H_2_O)		−0.110		−0.231^a^		0.024		−0.008		−0.004
**6**	V*P*		0.070		0.079		0.052		0.011		0.025
**7**	L*P*/*P*L/I*P*/*P*I	0.685^b^	0.224^a^	0.597^b^	0.145	0.459^b^	0.543^b^	0.630^b^	0.507^b^	0.526^b^	0.605^b^
**8**	*P*H/H*P*		−0.014		−0.024		0.005		0.049		0.037
**9**	F*P* (-H_2_O)	0.210^a^	−0.042	0.312^b^	−0.164	−0.124	−0.044	0.267^a^	0.076	0.257^a^	0.125
**10**	F*P*	0.662^b^	0.314^b^	0.554^b^	0.159	0.534^b^	0.587^b^	0.559^b^	0.509^b^	0.445^b^	0.571^b^
**11**	GP*P*	0.299^b^	0.246^a^	0.032	0.002	0.538^b^	0.350^b^	0.034	0.022	0.097	0.069
**12**	R*P*		0.417^b^		0.365^b^		0.539^b^		0.570^b^		0.506^b^
**13**	Unknown		0.064		0.051		0.040		0.145		0.139
**14**	GP*P*G	0.136	0.015	−0.077	−0.028	0.342^b^	−0.059	0.003	−0.034	0.124	0.052
**15**	PGP*P*G/GP*P*GP/PGGP*P*/*P*GPPG		0.282^b^		0.061		0.457^b^		0.212		0.271^a^
**16**	GP*P*GPT										
**17**	P*P*GP*P*GP/GP*P*GP*P*G		0.436^b^		0.166		0.626^b^		0.379^b^		0.425^b^
**18**	AGP*P*GP*P*GP/GP*P*GP*P*GPA/P*P*GP*P*GPAG		0.431^b^		0.242^a^		0.287^b^		0.536^b^		0.537^b^
**19**	A*P*GDRGE*P*GP*P*GP/*P*GDRGE*P*GP*P*GPA	0.542^b^	0.387^b^	0.374^b^	0.164	0.731^b^	0.716^b^	0.368^b^	0.495^b^	0.278^a^	0.514^b^
**20**	GP*P*GLAGP*P*GESGRE/P*P*GLAGP*P*GESGREG		0.328^b^		0.108		0.746^b^		0.387^b^		0.396^b^
**21**	*P*GFQGL*P*GPAGP*P*GE		0.515^b^		0.310^b^		0.549^b^		0.490^b^		0.449^b^
**22**	PAGR*P*GEVGPPGP*P*GPA	0.714^b^	0.425^b^	0.463^b^	0.196	0.783^b^	0.675^b^	0.508^b^	0.576^b^	0.379^b^	0.578^b^

Values show Pearson correlation coefficients (two-tailed analysis) from the denosumab trial dataset. “Serum” and “urine” sub-columns correspond to the peptide fragment biofluid. *N* = 89 serum samples, *n* = 94 urine samples. ^a^Correlation sig. at *p* < .05 level. ^b^Correlation sig. at *p* < .01 level.

In our analysis of the lung cancer dataset, Gly-Pro-Hyp and an unidentified compound with *m/z* 308.122 (compound 13; Table 1) were more abundant (*p* < .05, fold change >2) in urine from patients with bone metastasis; *n* = 24 patients with lung cancer and bone metastasis; *n* = 88 patients with lung cancer but without known bone metastasis (Figure 3E).

## Discussion

Our data show culturing osteoclasts on dentin substrate provides a useful tool to model bone resorption as it occurs in vivo. The diversity in peptide fragments observed, including oligopeptide products not previously reported, highlights the complexity of the bone resorption process extending beyond known proteolytic events. Twenty-one of the 22 dentin breakdown peptide products released into culture medium and discovered under controlled conditions in this model system were subsequently detected in human urine and/or serum. Analysis of the peptide fragment profiles in multiple clinical cohorts shows the potential for a subset of the molecules as new bone resorption markers. These candidate bone markers increased acutely in serum and/or urine following total hip replacement, in line with the marked peri-prosthetic bone loss previously reported in this cohort.^15^ Positive correlations were observed between the majority of peptide fragments and established clinical bone turnover markers, including serum and urine CTX. Our data strongly support that at least 4 peptide fragments are bone-derived, as these compounds showed clear decreases in patient serum, and 1 compound also in urine, following denosumab treatment (Figure 3B, Figure S5).

The peptide fragments we observed were rich in hydroxyproline residues and more frequently observed and at higher abundance in urine versus serum, consistent with previous studies.^3–8^ The products spanned a wide range in MW (174-1540 Da), from di-peptides to peptide fragments comprising 17 amino acid residues (Table 1). The lowest MW products, mainly di- and tri-peptides, mapped to various sequences across the collagen proteins present in dentin, with the exception of the di-peptides Phe-Hyp and Val-Hyp which were specific to COL1A1. In total, we identified 9 out of 22 peptides with sequences mapping exclusively to COL1A1, the protein that comprises 2 of the 3 chains in the type I collagen triple helix and the most abundant protein in dentin and bone. Among the COL1A1 specific products, 2 overlapped with the sequences of previously reported urine COL1A1 fragments and 1 reproduced exactly another previously reported fragment.^5^ Three of the COL1A1-specific resorption products are not previously described. Six of the seven highest MW products did not map to known COL1A1 protease cleavage sites, potentially indicating new COL1A1 proteolytic degradation pathways.

The peptide fragments we discovered indicate new cleavage sites in type I collagen. As shown in Figure 2, 8 out of 9 fragment sequences identified as COL1A1-specific did not overlap the positions of cleavage sites reported in MEROPS,^19^ one of the major protease databases. Instead, these peptide fragments mapped to more central positions within COL1A1 between the C- and N-terminal telopeptide sites at the extremities of the protein. Our observation adds to previous reports of type I collagen peptides resulting from unknown proteolytic events.^5^ Compared with previous analyses of urine peptides, we observed more low MW markers such as di- and tri-peptides. These smaller fragments were less specific, usually mapping to multiple sequences within COL1A1, including cathepsin K cleavage sites in addition to sequences within the other collagen proteins we considered (Table 1). Observation of small collagen fragments indicates our in vitro experiment procedure, involving analysis of extracellular media content after days of osteoclast activity, more represents end-stage resorption. Controlled digestion of type I collagen with cathepsin K in a previous study showed a chronology of proteolysis, with early cleavage events after 1 h of digestion occurring preferentially at the telopeptide cross-linking domains at the extremities of the type I collagen molecule. With increasing digestion time, up to 16 h, cleavage occurred across the breadth of collagen, including the central helical domain, producing peptide fragments comprising fewer than 4 amino acid residues.^10^

Our data support biofluid collagen peptide fragments as biomarkers in disorders affecting bone. In the Canadian total hip arthroplasty cohort, DXA previously showed the expected post-surgical loss of femoral bone mineral density proximal to prosthesis insertion and associated with increased fracture risk.^15^ In these patients we observed concordance between the longitudinal profiles of oligopeptide fragments and serum CTX and P1NP, with marked increases in peptide fragment abundance at the time point immediately following surgery. In the denosumab study, we showed that positive correlations with established bone biochemical markers were generally the most consistent for the COL1A1-specific peptide fragments. The 5 largest fragments identified in this study in addition to the dipeptide Phe-Hyp, all COL1A1-specific, showed statistically significant correlations with serum P1NP, serum CTX, urine alpha-CTX, and urine beta-CTX (Table 2). These significant correlations held whether the peptide fragments were detected in serum or urine. The response of peptide fragments to denosumab is probably underestimated in this dataset due to the relatively small scale of the study, limiting statistical power. Nevertheless, the data strongly support the largest peptide fragment identified in this analysis (compound 22, Table 1) as an excellent candidate for future exploration as a new bone biomarker. This molecule is COL1A1-specific, acutely increased immediately following total hip replacement (Figure 3A), decreased in serum and urine following denosumab treatment (Figure 3B) and correlates with all established bone biomarkers investigated in this study (*p* < .05; Figures 4B and 5B, Table 2), with exception of urine TRAP.

Our finding of elevated urine bone peptide fragments in bone metastasis is consistent with data on type I collagen markers in bone cancer.^20–22^ An osteolytic phenotype is frequently induced by presence of bone tumors, associated with stimulation of osteoclastogenesis through RANKL expression and cathepsin K activity.^23–25^ In our lung cancer cohort, we previously showed changes in bone peptide fragments in the last weeks of life.^17^ Two of the COL1A1-specific collagen peptides reported here progressively increased in urine leading up to death, from greater than 3 mo up to the last week before death; these were the dipeptide Phe-Hyp and the highest MW COL1A1 fragment observed here (compound #22; Table 1). Another unidentified bone marker (compound #13; Table 1) decreased leading up to death. These findings provide major insights into the fundamental biological changes that occur at the end of life, implicating divergent bone resorption pathways as part of a multifactorial process of dying.

Quantification of global biofluid type I collagen peptide fragments has potential to provide a more detailed assessment of bone metabolism than established bone turnover markers. Established bone resorption markers are products of cathepsin K. We show that many bone breakdown products result from collagenolytic activity of other enzymes including MMPs. Altered activity of specific MMPs is a major factor in the pathogenesis of various common diseases associated with bone dysregulation, including osteoarthritis, bone cancer, and a range of conditions associated with osteolysis.^26^ It is worth noting the peptide fragments observed in these patients that showed longitudinal changes inconsistent with CTX and P1NP despite being demonstrated as bone breakdown products in osteoclast cultures. Serum Leu-Hyp (Figure S4), for example, decreased following total hip replacement. The reason for this finding is unknown but suggests a multiplicity of bone resorption pathways revealed by monitoring the range of peptide fragments presented here. It is also possible that some peptide fragments observed in biofluids in vivo do not exclusively arise from bone collagen or from osteoclastic enzyme activity. Dietary protein and breakdown of connective tissues other than bone may contribute to the biofluid pools of some peptide fragments. MMPs of non-osteoclast origin, for example, MMP13 produced by periosteoclast cells, can also degrade bone collagen.^27^

Previous analyses of type I collagen fragment peptides in urine^4–8^^,^^28^ or from in vitro digestion of human type I collagen protein by cathepsin K^10^ show considerable variation in the fragments observed. Many factors are likely to account for this variation, including the experimental conditions used in studies of bone or collagen digestion in vitro, in addition to instrumentation and analytical conditions and workflows employed in detection of peptides. The LC/MS technique used here is different to platforms used in other studies reporting collagen peptide fragments, which have frequently used capillary electrophoresis coupled to mass spectrometry.^4–8^^,^^28^ Our LC/MS approach has been employed extensively in small molecule analyses including metabolomic applications and is possibly more optimal for detection of low, versus high, MW peptides.^13^ This contrasts the proteomic approaches used in previous studies and shows the potential to detect low-medium MW bone collagen fragments in serum and urine metabolomic datasets. The data mining approach in terms of initial molecular feature extraction on raw LC/MS data was untargeted, in contrast to other studies reporting collagen-derived peptides. Identification of molecules of interest (ie, bone resorption products) was performed post hoc. In this way, the extraction of signals/peptide features did not depend on existing knowledge including the sequences of known peptides and data from existing peptide libraries. In other words, the profiles of ion features that were detected and extracted from the raw data, independent of their subsequent biochemical identifications, were included in the analysis. An advantage of this untargeted approach is that some peptide features remain incompletely characterized and are therefore not detectable using common proteomic data mining approaches which rely upon existing peptide libraries. For type I collagen, incompletely characterized features include the extent of cross-linking, known to occur at multiple telopeptide and helical regions, and post-translational modifications.^10^

In conclusion, our data demonstrate the powerful approach of untargeted high-resolution MS analysis for characterization of the range of type I collagen degradation products. The overlap between collagen fragments observed in osteoclast cultures and in human serum and urine shows culturing of osteoclasts on dentin is relevant to identify potential bone resorption markers. The novel collagen fragments presented, some of which are COL1A1-specific, provide new insights into the pathways of collagen degradation and support the clinical potential of monitoring collagen peptide fragments in bone disorders.

## Supplementary Material

Supplementary_revised_Jul25_ziaf160
