# Supplementary material for: Identification of novel collagen breakdown products by human osteoclasts in vitro and in vivo
Source: JBMR Plus. 2025 Oct 11;9(12):ziaf160. doi: 10.1093/jbmrpl/ziaf160 (PMC12596726; doi:10.1093/jbmrpl/ziaf160)
Supplement: Supplementary_revised_Jul25_ziaf160 [file supplementary_revised_jul25_ziaf160.docx]

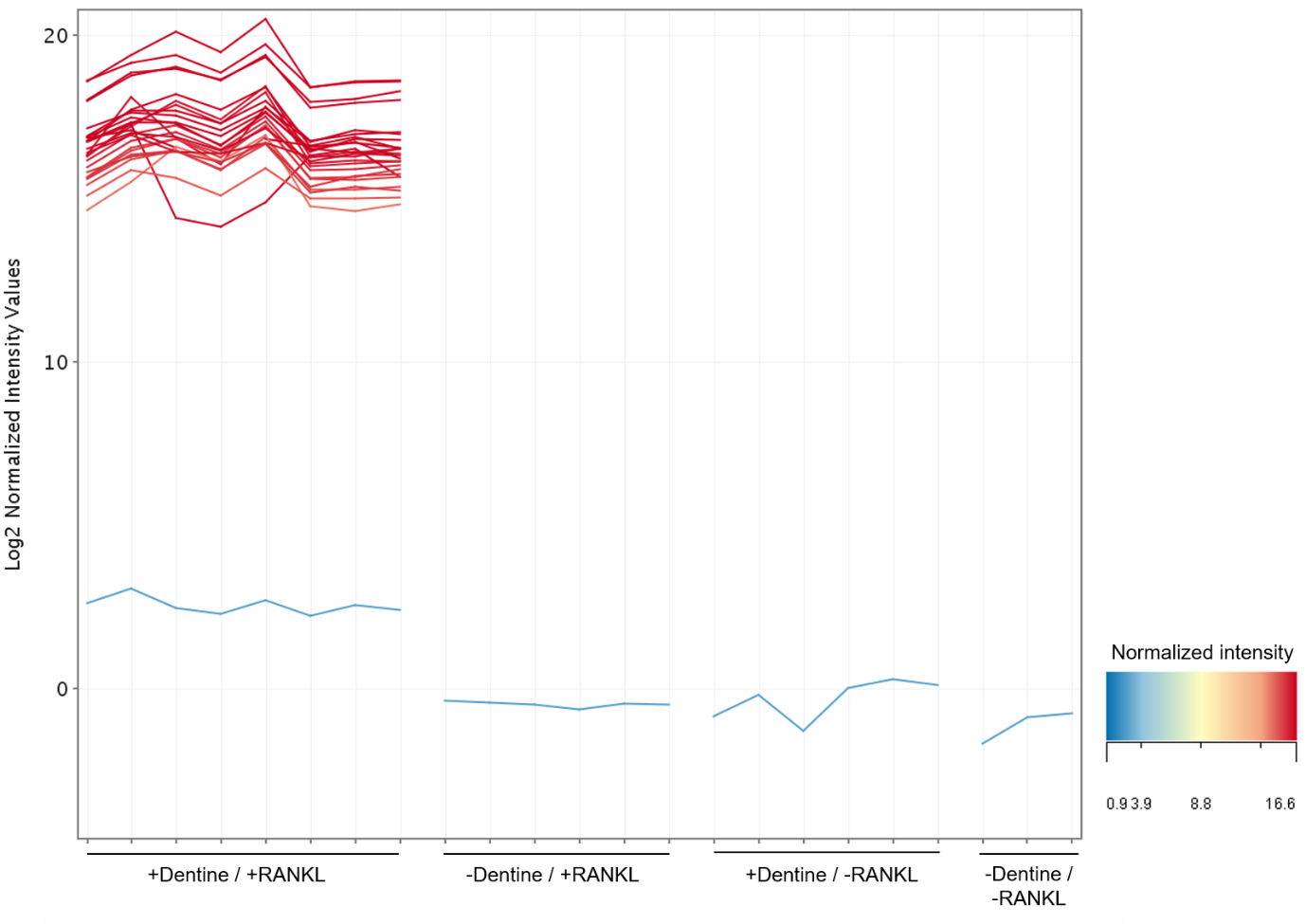
**Figure S1**. Profile plot showing extracellular media LC/MS peak area abundances of the 22 bone resorption products across the *in vitro* experiment conditions. Each line represents one compound. A clear cluster of compounds was observed representing products only detected in resorptive conditions (+dentine / +RANKL cultures); red profiles, top left portion of plot. The tripeptide Gly-Pro-Hyp (blue profile, lower portion of plot) was detected in all conditions, but with markedly greater abundance in +dentine / +RANKL compared with the three control groups.

**Figure S2**. Peak area abundance values for hyp-his (or his-hyp); the oligopeptide fragment observed in the dentine slice assay but without inhibition by zoledronic acid (Zol).


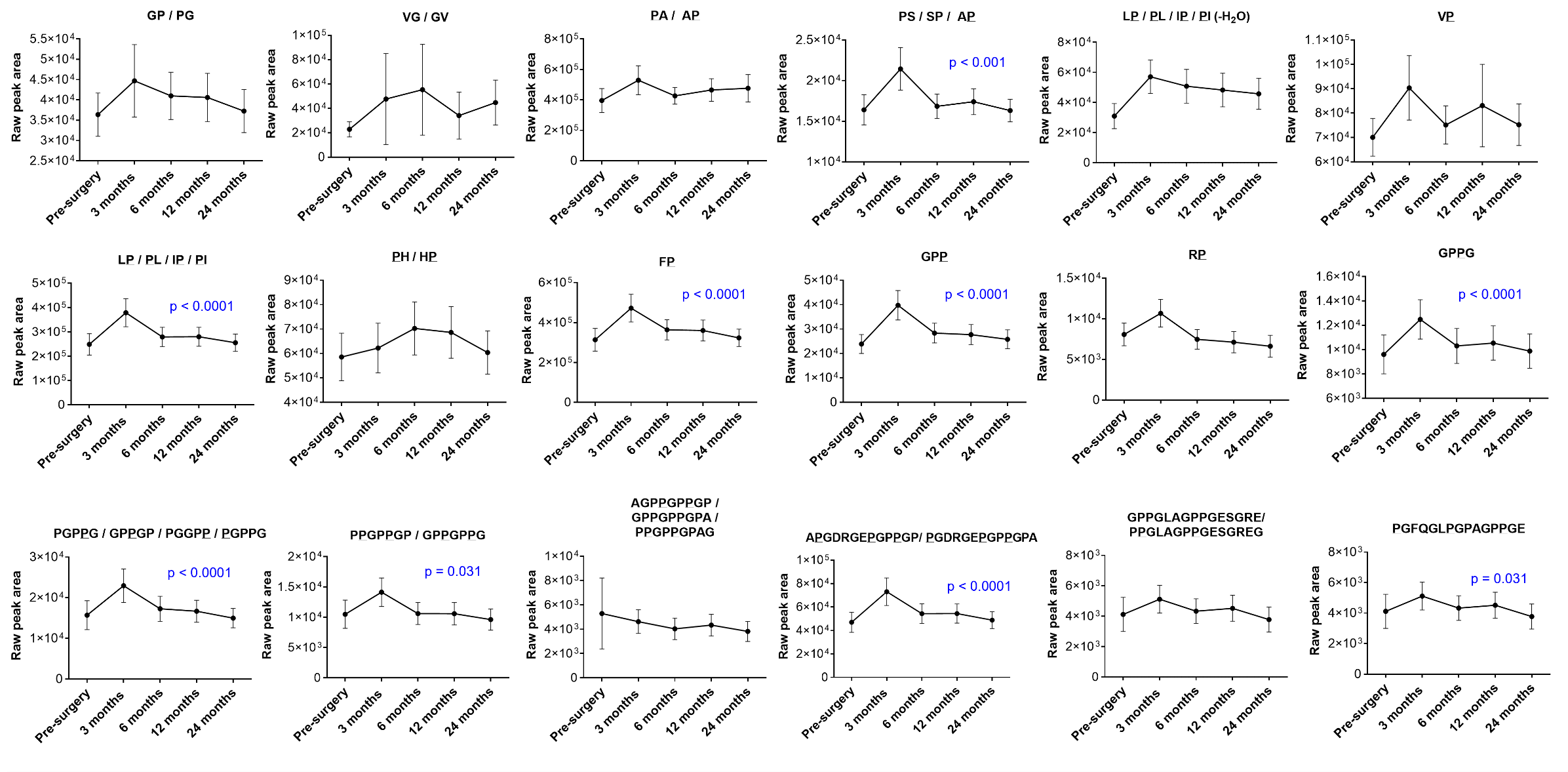


**Figure S3.** Urine data from the Canadian total hip arthroplasty cohort.^1^ Mean abundance profiles of oligopeptide bone resorption products detected in urine collected from patients pre-surgery, then at 3, 6, 12 and 24 months post-surgery. P-values are from one-way repeated measures ANOVA. Error bars represent 95 % confidence intervals. Note urine profile of compound 22 in this dataset is shown in Figure 3.

^1^Slullitel et al. J Bone Joint Surg Am. 2021;103(1):74-83. doi:10.2106/JBJS.20.00351


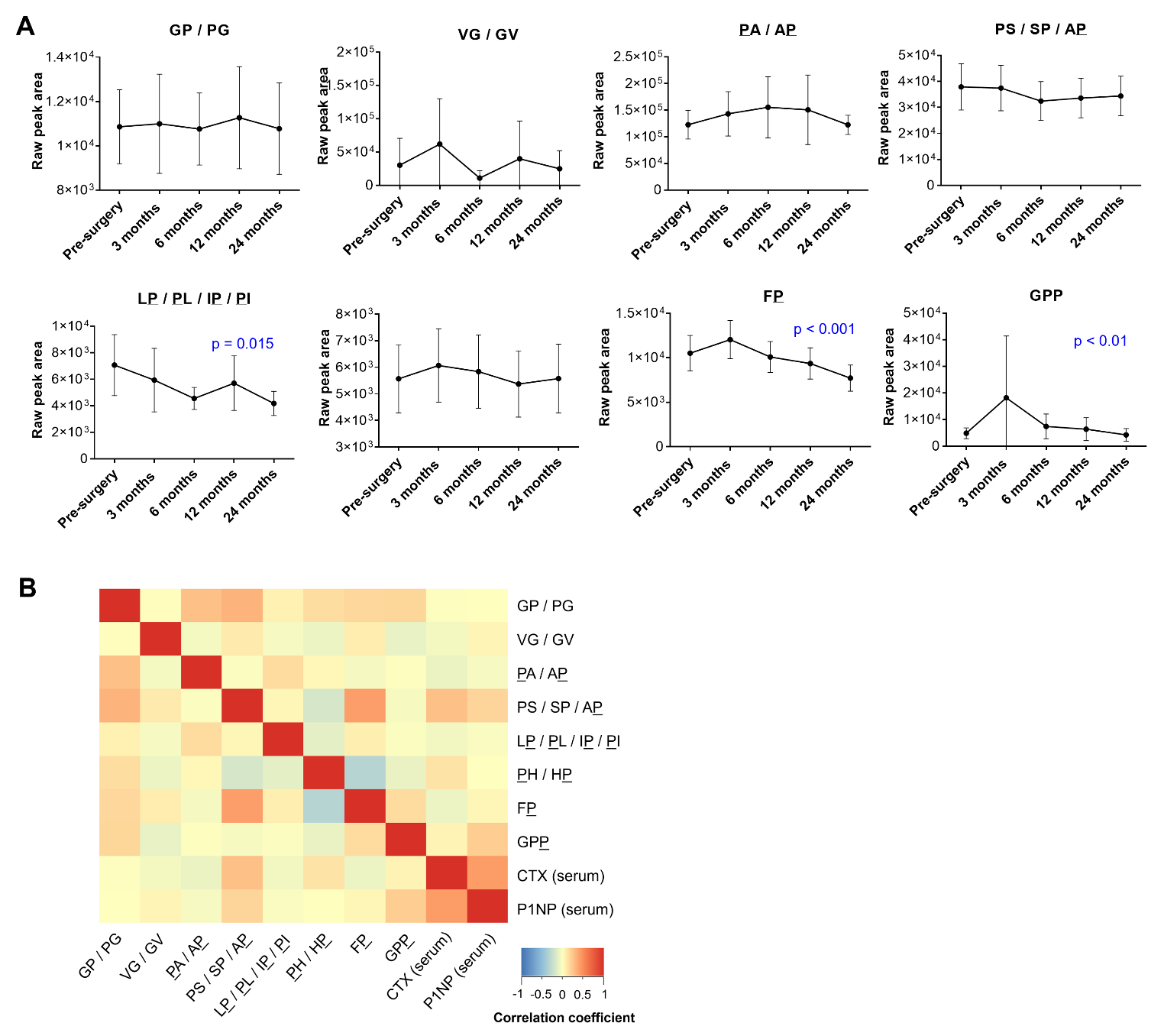


**Figure S4.** Serum data from the total hip arthroplasty cohort.^1^ **A:** Mean abundance profiles of oligopeptide bone resorption products detected in urine collected from patients pre-surgery, then at 3, 6, 12 and 24 months post-surgery. P-values are from one-way repeated measures ANOVA. Error bars represent 95 % confidence intervals. **B:** Heatmap from Pearson’s correlation analysis of data from serum oligopeptide compounds in addition to serum CTX and P1NP. ^1^Slullitel et al. J Bone Joint Surg Am. 2021;103(1):74-83. doi:10.2106/JBJS.20.00351


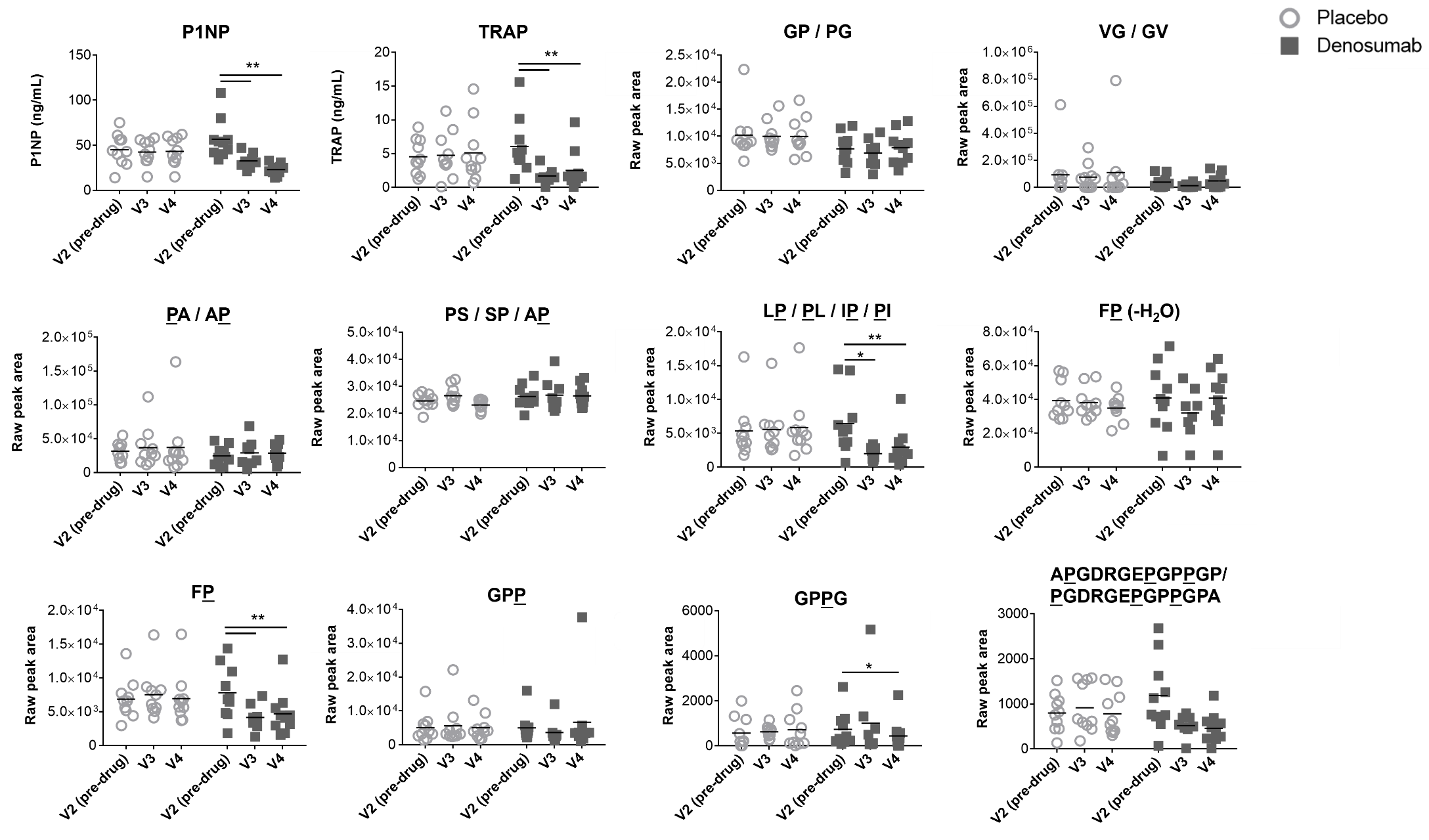


**Figure S5.** Serum data from the denosumab study.^2^ Serum biochemical abundance profiles of peptide fragments in patients on denosumab/placebo at 4 (visit 3) and 8 weeks (visit 4) on treatment versus baseline (visit 2). P-values are from repeated measures ANOVA on patients (serum data, denosumab n = 8 patients, placebo n = 11 patients; urine data, denosumab n = 7 patients, placebo n = 9 patients) with samples available for all three time points. Dot plots show data from all samples analysed (serum, n = 58; urine, n = 58). *p <0.05, **p <0.01; Benjamini-Hochberg false discovery rate adjusted.

^2^Mahatma et al. Lancet Rheumatol. 2021;3(3):e195-e203. doi:10.1016/S2665-9913(20)30394-5

**Table S1.** Accurate mass and retention time data for bone breakdown products identified.

| Peptide | Measured *m/z* | Retention time (minutes) | Charg*e* (*Z*) |
| --- | --- | --- | --- |
|  |  |  |  |
| GP / PG | 173.0925 | 1.6 | 1 |
| VG / GV | 175.1102 | 1.89 | 1 |
| PA / AP | 185.0926 | 2.58 | 1 |
| PS / SP / AP | 203.1032 | 1.36 | 1 |
| LP / PL / IP / PI | 227.1392 | 5.97 | 1 |
| VP | 231.1344 | 1.93 | 1 |
| LP / PL / IP / PI | 245.1501 | 3.29 | 1 |
| PH / HP | 251.1142 | 1.54 | 1 |
| FP | 261.1245 | 6.4 | 1 |
| FP | 279.1345 | 4.25 | 1 |
| GPP | 286.1431 | 1.74 | 1 |
| RP | 288.1674 | 1.27 | 1 |
| Unknown | 308.122 | 1.73 | 1 |
| GPPG | 343.1619 | 1.73 | 1 |
| PGPPG / GPPGP / PGGPP / PGPPG | 440.2132 | 3.78 | 1 |
| GPPGPT | 541.2603 | 3.76 | 1 |
| PPGPPGP / GPPGPPG | 610.282 | 3.78 | 1 |
| AGPPGPPGP /  GPPGPPGPA /  PPGPPGPAG | 389.6906 | 4.71 | 2 |
| APGDRGEPGPPGP/ PGDRGEPGPPGPA | 626.2847 | 4.44 | 2 |
| GPPGLAGPPGESGRE/  PPGLAGPPGESGREG | 705.3379 | 5.95 | 2 |
| PGFQGLPGPAGPPGE | 713.3364 | 5.6 | 2 |
| PAGRPGEVGPPGPPGPA | 771.3841 | 5.92 | 2 |
